# Subsidence and Clinical Impact of Obesity in Short-Stem Total Hip Arthroplasty Using a Collarless, Triple-Tapered, Cementless Stem

**DOI:** 10.3390/jcm13247596

**Published:** 2024-12-13

**Authors:** Michael Stephan Gruber, Johannes Schöning, Martin Bischofreiter, Harald Kindermann, Arndt-Peter Schulz, Nico Hinz, Emanuel Mayböck, Reinhold Ortmaier

**Affiliations:** 1 Department of Orthopedic Surgery, Ordensklinikum Linz Barmherzige Schwestern, Vinzenzgruppe Center of Orthopedic Excellence, Teaching Hospital of the Paracelsus Medical University, 5020 Salzburg, Austria; 2 Medical Faculty, Johannes Kepler University Linz, Altenbergerstraße 69, 4040 Linz, Austria; 3 Department of Marketing and Electronic Business, University of Applied Sciences Upper Austria, 4400 Steyr, Austria; 4 Zentrum für Klinische Forschung, BG Klinikum Hamburg, 21033 Hamburg, Germany; 5 Section Medicine, Universität zu Lübeck, 23538 Lübeck, Germany; 6 Department of Orthopedic Surgery, Traumatology & Sports Medicine, BG Klinikum Hamburg, Bergedorfer Str. 10, 21033 Hamburg, Germany

**Keywords:** short-stem total hip arthroplasty (THA), obesity and THA, postoperative subsidence in THA, clinical outcomes in hip arthroplasty, stem size and subsidence correlation

## Abstract

**Background:** Short-stem total hip arthroplasty (THA) has gained popularity due to its bone-sparing technique, but its outcomes in patients with obesity remain uncertain. The aim of this study was to investigate the impact of obesity on postoperative subsidence and clinical outcomes after short-stem THA. **Methods:** A retrospective cohort study with a minimum follow-up of 24 months was conducted on 163 patients who underwent short-stem THA with a collarless, triple-tapered, cementless stem achieving fixation in the metaphyseal region. Patients were categorized into obesity (Body Mass Index, BMI ≥ 30 kg/m^2^) and nonobesity (BMI < 30 kg/m^2^) groups. Subsidence rates, clinical outcomes, and complications were analyzed to assess the influence of BMI on the outcome of short stem THA. Regression analysis was performed to assess the influence of the independent variables (BMI, stem size, deviation from planning) on subsidence. **Results:** The obesity group (mean follow-up 58.6 months) exhibited significantly greater subsidence rates than did the nonobesity group (mean follow-up 38.9 months; 2.6 mm vs. 2.2 mm, *p* = 0.015). After removal of outliers, regression analysis revealed no linear relationship between BMI and subsidence (*p* = 0.35), but planned stem size was significantly correlated with subsidence (*p* = 0.005). Moreover, patients with obesity and larger planned stem sizes experienced greater subsidence. Clinical outcomes improved significantly in both groups. **Conclusions:** Obesity is associated with increased subsidence in short-stem THA, particularly in patients with larger planned stem sizes. Although BMI alone may not predict subsidence, careful selection of stem size and precise imaging techniques are crucial for minimizing subsidence risk in patients with obesity.

## 1. Introduction

Short-stem total hip arthroplasty has become increasingly popular in the last decade [1,2,3]. The mean age of patients who underwent artificial joint arthroplasty has recently decreased. At present, more than 20% of patients who receive total hip arthroplasty (THA) are younger than 60 years [4,5]. Consequently, the length of time a prosthesis is worn is increasing, and as a result, the likelihood of having to replace the prosthesis is also increasing. Short-stem THA has been developed as a bone-preserving alternative to conventional THA, particularly suited for younger and more active patients. The primary rationale for short-stem implants lies in their metaphyseal fixation, which minimizes stress shielding and preserves proximal femoral bone stock. This approach is particularly advantageous in facilitating future revisions by maintaining sufficient bone reserves and reducing the risk of intraoperative complications during re-implantation [6]. In contrast to the age of patients undergoing THA, the average body mass index (BMI) of the population in the global West has increased substantially in recent decades [7,8]. Obesity is defined as a “BMI greater than or equal to 30” [9].

Some studies assumed that associated increased mechanical stress leads to impaired osteointegration of the prosthesis in the period between primary and secondary fixation. This represents a potential risk for increased subsidence, which is considered a risk factor for aseptic prosthesis loosening in conventional stems [10,11].

While studies on the mid-term outcome in a general patient cohort have provided excellent results in terms of clinical and radiological outcomes, as well as the complication rate of short-stem THA, data on its use in patients with obesity are still rare and not sufficiently conclusive. Some studies have revealed no evidence of increased subsidence in patients with obesity, while other studies have shown contrary trends [6,12,13,14,15,16,17,18].

In this context, our underlying research question was whether BMI has an influence on postoperative subsidence and clinical outcomes after short-stem THA, with one obesity cohort (BMI ≥ 30 kg/m^2^) and one nonobesity cohort (<30 kg/m^2^). In contrast with the existing literature, this study solely focused on overweight patients with a matched control cohort, including a large population of patients with a BMI ≥ 30 kg/m^2^. Furthermore, a focus was set on the analysis of stem sizes.

## 2. Materials and Methods

This study was approved by the ethics committee of the Johannes Kepler University Ethics Commission (1258/2023 on 15 November 2023) and conducted in accordance with the principles of the Declaration of Helsinki. The Strengthening the Reporting of Observational Studies in Epidemiology (STROBE) guidelines were followed. The study was registered with ClinicalTrials.gov (NCT06430723).

Results from routinely performed tests and X-ray scans from 200 patients were analyzed, focusing on clinical outcome and radiographic analysis of the subsidence of the stem in patients with obesity. All surgeries were performed in a single institution by different surgeons. Patients were classified into two groups based on BMI: patients with obesity (BMI ≥ 30 kg/m^2^) and nonobese patients (BMI < 30 kg/m^2^) [9]. The primary outcome was defined as subsidence of the stem and secondary outcomes were defined as clinical outcome (Harris Hip Score, HHS) and complication rate.

**Figure 1:**
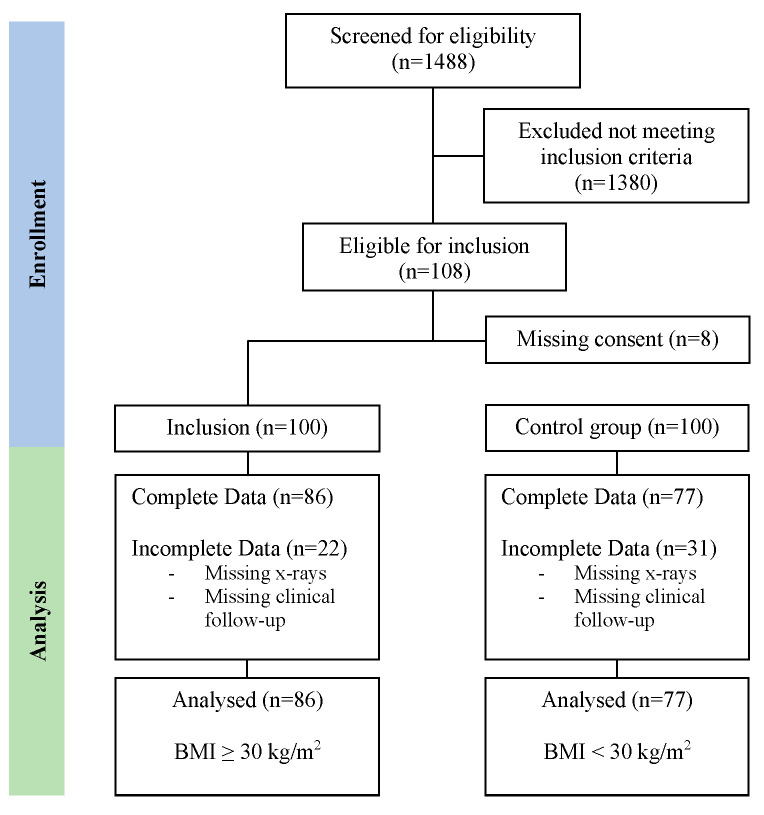
Flow chart of the inclusion process.

The inclusion criteria were (1) patients who underwent primary short-stem THA with a Mathys optimys short stem between 1 January 2018 and 31 December 2020, (2) available preoperative and postoperative radiographs for the assessment of subsidence, (3) a minimum BMI of 30 kg/m^2^ for the obesity group, and (4) a minimum follow-up of 24 months. The exclusion criteria were (1) previous surgeries for the hip in question, (2) incomplete clinical data or (3) incomplete radiological data. One hundred patients were initially included in the obesity cohort, and one hundred patients were included in the control group (BMI < 30 kg/m^2^) (Figure 1).

The clinical evaluation included patient demographics, comorbidities, surgical details (including implant size and placement technique), postoperative complications and the Harris Hip Score. Radiographic analysis focused on measuring stem subsidence, defined as the axial displacement of the stem in relation to the femoral neck. This was measured using the EBRA-FCA-Method (Einzel-Bild-Roentgen-Analyse Femoral-Component-Analysis, Institute for Basic Engineering Sciences, University of Innsbruck, Innsbruck, Austria; https://www.uibk.ac.at/en/projects/ebra/, accessed on 1 August 2023), with a minimum of three necessary radiographs [19,20]. The immediate postoperative radiographs were compared to two radiographs at follow-up, with a minimum of 24 months from surgery to follow-up for the most recent image.

The planned stem size data were retrieved from the planning records, which are routinely recorded via mediCAD (https://medicad.eu, accessed on 1 October 2024) at this institution. The planning is performed following the fit-and-fill principle. The stem size used was extracted from surgical reports.

Blood loss was calculated according to Bourke and Smith [21,22]:

BL = BV × (Hct_0_ − Hct_t_) × (3 − Hct_mean_)

BL, blood loss
BV, blood volume
Hct_0_, preoperative hematocrit
Hct_t_, postoperative hematocrit
Hct_mean_, mean pre- and postoperative hematocrit

Adverse postoperative events were documented using the Clavien-Dindo classification.

### 2.1. Data Analyses

Complete data were available for 164 of the 200 patients included in this study (86 in the obesity group and 77 in the nonobesity group).

All parameters collected from these patients were first analyzed descriptively, using various graphs and tables to provide a comprehensive overview of the characteristics of the included patient cohort. Due to the presence of outliers, nonparametric tests, such as the Mann–Whitney U test (MW-T), were used to test the impact of BMI on subsidence.

**Figure 2:**
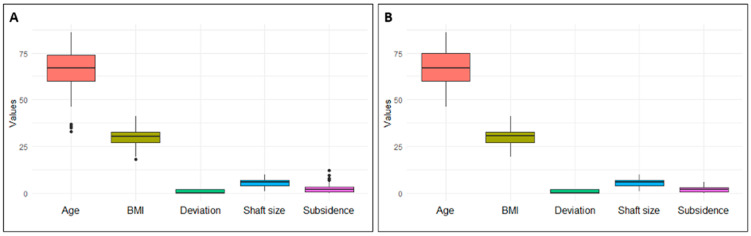
Outliers were identified via boxplots (**A**) and subsequently removed (**B**).

Given the exploratory nature of the study, an additional regression analysis was performed to examine the influence of the independent variables (IVs)—age, BMI, socket size, and deviation—on subsidence (the dependent variable; DV), to explore if there was any impact on subsidence. Before performing the regression analysis, any existing outliers in all metric variables were first identified using boxplots so that they could be removed (see Figure 2). In this way, the assumptions required for the regression analysis were largely fulfilled. This resulted in a total of 145 patient records being used for this analysis. The deviation from the planned stem size (deviation) was dummy-coded (0 = no deviation from plan) prior to the regression analysis. Due to the 6 × 2 table, postoperative complications were evaluated using a Fisher–Freeman–Halto test—a chi-square test was not performed, because it lacks reliability if the expected frequencies in some cells are small.

In addition, the effect of BMI on secondary outcomes was examined in the same two groups as previously mentioned (BMI < 30 and BMI ≥ 30). Differences in the secondary outcomes between these two groups were again analyzed using the MW-T test for continuous variables and the chi-squared test for categorical variables. This methodological approach was chosen to ensure a comprehensive and robust analysis of the relationships between BMI and postoperative subsidence, as well as other relevant outcome measures. Analyses were performed using R version 4.2.2.

### 2.2. Demographic Analyses

Table 1 shows the demographic variables of the obesity and nonobesity groups. There was no statistically significant difference concerning age or sex. The minimally invasive anterolateral approach was performed in all patients, and staples were used for skin closure in each patient. The mean follow-up time was 38.9 months for the nonobesity cohort and 58.6 months for the obesity cohort.

## 3. Results

**Figure 3:**
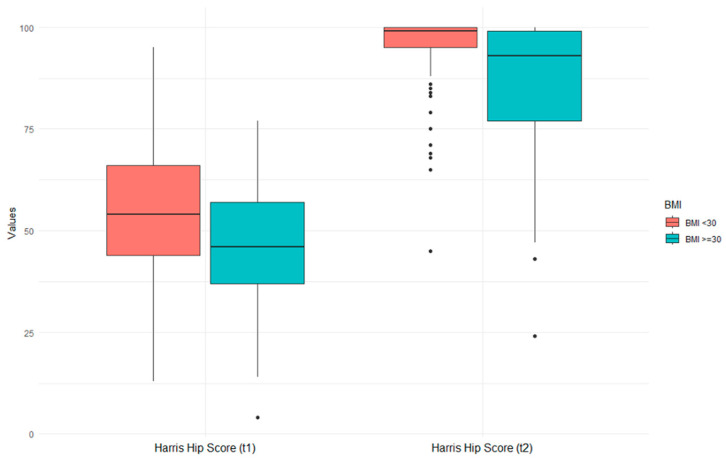
BMI and its effect on preoperative and postoperative Harris Hip Scores. While the left image (t1) shows preoperative HHS, the right image (t2) shows postoperative HHS.

A comparison of the two groups revealed a significantly greater rate of subsidence in the obesity group than in the nonobesity group (Table 2). The obesity group had a subsidence rate of 2.6 mm compared to 2.2 mm in the nonobesity group (W = 2475.5, *p* = 0.015). In addition, the postoperative HHS was lower for the obesity group than for the nonobese group (Figure 3).

The regression model revealed that 13% (adjusted R^2^) of the variance was explained by obesity status (Table 3). Contrary to the results from the MW-T, BMI, as a continuous parameter, had no significant linear influence on subsidence upon the exclusion of outliers.

However, a significant correlation between the planned stem size and the rate of subsidence in both the obesity and nonobesity groups was found (Table 3). Specifically, patients with larger planned stem sizes experienced greater subsidence in short-stem THA (*p* value = 0.005).

The evaluation of planned compared to used stem size revealed greater precision of the planning in the nonobesity group (Table 4). Furthermore, the use of a larger stem compared to the planned stem size was significantly linked with greater subsidence (Table 3, deviation, *p* value = 0.005).

Postoperative complications were evaluated using the Clavien–Dindo classification. The groups were similar, except for grade 4 (obesity cohort: *n* = 5; nonobesity cohort: *n* = 0; Table 5). However, the Fisher–Freeman–Halto test showed no significant difference (*p* = 0.284). In the obese group, there were two revisions, one each due to aseptic loosening and subsidence (2/86, 2.3%), but no further subsidence-related complications were noticed. One patient underwent revision in the nonobese group due to aseptic loosening of the acetabular cup (1/77, 1.3%), and one patient suffered from a single dislocation, which did not require revision surgery.

## 4. Discussion

Our study focused on subsidence associated with short-stem THA and its association with obesity. The patient cohort comprised 163 patients, of whom 86 were classified as having obesity and 77 were classified as nonobese. The primary outcome of this study was defined as stem subsidence because it represents a critical biomechanical parameter that reflects the initial stability and long-term integration of the prosthesis. Secondary outcomes were defined as clinical outcomes, measured using the Harris Hip Score (HHS), and complication rates. These outcomes were chosen to capture the broader impact of obesity on patient recovery and prosthesis functionality. The HHS is a widely validated tool that evaluates pain, function, and mobility, directly linking subsidence to patient-centered outcomes. Complication rates, categorized by the Clavien–Dindo classification, were included to identify any obesity-related risks affecting perioperative or postoperative safety. The clinical relevance of this design lies in its comprehensive approach: subsidence highlights biomechanical performance, while secondary outcomes provide a holistic view of the patient experience and safety profile. This combination ensures that the study addresses both the technical and practical implications of short-stem THA in obese compared to non-obese populations.

The results of our study demonstrated a significant difference in the rate of subsidence between the obesity and nonobesity groups (BMI ≥ 30 kg/m^2^: mean 2.6 mm, SD = 2.6 mm; BMI < 30: mean 2.2 mm, SD = 2.1; *p* = 0.015) after 36 months of follow-up. The worse absolute postoperative HHS score of patients in the obesity group may lead one to think that the 0.4 mm difference in subsidence between the two groups represents a significant clinical difference. However, the obesity group already showed worse Harris Hip Scores preoperatively. The absolute difference in HHS between the groups decreased from preoperatively (11.5 points) to postoperatively (8.5 points). This suggests that the greater subsidence has no clinical significance based on HHS. Further evaluation using regression analysis revealed no linear relationship between BMI and subsidence (*p* = 0.35), indicating that the impact of obesity on subsidence is not solely dependent on BMI. Other factors may include weight, gender, and DORR configuration of the femur.

The literature is divided about the effects of BMI on subsidence, as some studies have highlighted a possible association between obesity status and an increased risk of experiencing subsidence in THA [3,4,24,25], while most others did not [5,6,7,8,17,26,27,28,29]. For example, a recent study by Saracco et al. [26] showed that there was no correlation between obesity status and the risk of experiencing subsidence [6]. However, they focused only on patients with obesity (BMI ≥ 30), and the results were not compared with those of a control group. Contrary to these findings, Kutzner et al. [13] reported that male patients with obesity had a greater initial rate of subsidence than nonobese patients following THA with an optimys short stem, but no significant differences were reported [10]. A mean subsidence of 1.38 mm and 1.56 mm was measured in patients with a BMI < 30 kg/m^2^ versus those with a BMI ≥ 30 kg/m^2^, respectively (*p* = 0.22) (2016). A study from 2020, also conducted by Kutzner et al. [14], showed a mean subsidence of 1.43 mm (BMI < 30 kg/m^2^) and 1.71 mm (BMI ≥ 30 kg/m^2^) (*p* = 0.261). Neither study showed a significant correlation between BMI and subsidence. Furthermore, Schaer et al. were unable to show a significant correlation in their study within a 5-year observation period (2.14 mm for BMI < 30 kg/m^2^; 1.8 mm for BMI ≥ 30 kg/m^2^) (*p* = 0.6) [11].

However, Freitag et al. reported a trend toward increased subsidence rates in patients with a BMI ≥ 30 kg/m^2^ (*p* = 0.25) who underwent Fitmore short-stem THA [16]. Our results thus support the assumption that a BMI ≥ 30 kg/m^2^ and the associated higher weight of the patients can lead to greater micromovements at the bone–implant interface and subsequently delay the achievement of secondary stability, resulting in increased subsidence. However, it is important to point out that this study did not analyze the osteointegration of the stem. Another study that showed a tendency toward increased migration in overweight patients was performed by Afghanyar et al. in 2020 [30]. They also reported initial migration with subsequent stabilization.

Our findings align closely with previous research, which has revealed a strong association between the use of larger stem sizes (6 or greater) and increased subsidence, particularly among female patients. Schaer et al. posited that this may be due to the tendency to use smaller implants in women, driven by the fear of intraoperative periprosthetic fractures [11]. Notably, our study corroborates these conclusions, demonstrating a significant correlation between stem size and subsidence.

Interestingly, the preference for larger stems in patients with a BMI ≥ 30 kg/m^2^ may stem from the surgeon’s perception of the need for enhanced stability or concerns about potential complications with smaller implants in this patient population. However, the underlying reason for the discrepancy between the planned and actual stem sizes used in obese individuals remains unclear. This discrepancy may be attributed to inaccuracies in the X-ray calibration during preoperative planning, a consequence of the patient’s obesity.

To address this issue, it is crucial for surgeons to employ precise imaging techniques and calibrations when planning short-stem total hip arthroplasty in patients with obesity, rather than relying solely on preoperative planning. A study by Reinbacher et al. showed that increased BMI leads to inaccurate 2D planning of the stem (*p* = 0.041) [18]. And while 87% of stems were accurate within one size in a population followed up by Holzer et al., the accuracy was significantly lower in overweight patients [31].

We recommend considering intraoperative imaging for patients with a BMI ≥ 30 kg/m^2^ to ensure accurate stem size selection and mitigate the risk of subsidence. Furthermore, implementing preventative measures, such as reduced weight bearing in the immediate postoperative period, may help promote proper healing and stability of the implant in obese patients undergoing short-stem THA. However, this hypothesis requires further investigation and evaluation.

The clinical results showed significant improvement in both the nonobesity and obesity patient cohorts. Contrary to a study by Gabrion et al. from 2023 [12], BMI seems to influence clinical outcome to some extent. Their study reported an HHS of 95.7 points for patients with a BMI < 30 kg/m^2^ and 92.9 points for patients with a BMI ≥ 30 kg/m^2^. Our results indicate generally worse HHS in the obesity group, while the absolute difference between both groups decreased postoperatively. While the cohort with a BMI < 30 kg/m^2^ improved from a preoperative HHS average of 56.5 points (SD = 19.2) to a score of 94.1 points (SD = 10.4, *p* < 0.001), the HHS of patients with a BMI ≥ 30 kg/m^2^ improved from 45.5 points (SD = 16.0) to 85.6 points (SD = 17.1, *p* < 0.001). The absolute difference between both groups decreased from 11.5 points preoperatively to 8.5 points postoperatively. According to the Harris Hip Score, the results of the nonobese group were considered excellent, and the results from the obese group were considered good [11]. Unfortunately, Gabrion et al. did not report the preoperative values, so this aspect of the relative increase of HHS cannot be compared [12]. Hinz et al. reported similar results to our study in a cohort of 224 patients and a comparison between patients with obesity and nonobese patients [32]. They also found no differences in the complication rate between the two groups. In our study cohort, there were two revision surgeries in the obesity cohort, one due to aseptic loosening of the stem after 2 years and one due to subsidence. One patient of the nonobesity cohort required revision surgery due to loosening of the acetabular cup, and one patient suffered from a single dislocation of the hip, which did not require surgical treatment. There were no further subsidence-related complications. Overall, the postoperative complications were comparable, but the obesity group had more severe complications. However, the difference in frequency was not statistically significant (*p* = 0.284). Notably, none of the nonobese patients needed intensive care after surgery, while five of the patients in the obesity cohort needed postoperative intensive care. This leads to the assumption that obesity is a risk factor for postoperative intensive care after surgery.

Given the implications of obesity for hip arthroplasty surgery, it is essential to explore strategies for managing THA challenges in individuals with obesity. Barrett et al. reviewed the literature in 2018 and discussed the risks associated with overweight, such as a higher complication rate and a slightly worse clinical outcome [33]. Considering the significant impact of obesity on subsidence in short-stem THA, future studies should focus on preventative measures for subsidence in THA for patients with obesity. By identifying and implementing effective preventative strategies, such as specialized implant designs, surgical techniques, or postoperative partial weight bearing, the overall success and longevity of short-stem hip implants in individuals with obesity may be optimized.

The literature emphasizes the importance of tailored approaches to address the unique challenges presented by obesity in THA, and our study contributes to this body of knowledge by providing valuable insights into the relationship between obesity and subsidence in short-stem hip implants.

## 5. Limitations

This study has several limitations. First, this is a single-institution approach, which could lead to a selection bias due to uniform surgical technique of few highly experienced surgeons. However, the patients were treated by surgeons with different levels of training, which decreases the selection bias. It would be better, though, to perform a standardized follow-up in a multi-center setting. Second, the patients were only categorized into BMI < 30 kg/m^2^ or ≥30 kg/m^2^. The classification of obesity by the WHO states more subgroups; however, an adequate patient count per group has to be reached to achieve a sound analysis. Therefore, future studies should take careful patient selection according to classification of obesity into account. Third, other confounding factors such as smoking or quality of bone stock measured with Dual-Energy X-ray Absorptiometry or similar methods were not evaluated. Since these factors may present important differential reasons for subsidence, they should be evaluated separately in a follow-up study, most appropriately in a prospective setting.

## 6. Conclusions

In conclusion, while there is no direct linear relationship between BMI and subsidence in short-stem THA, our study revealed that patients with obesity with larger planned stem sizes are at greater risk of experiencing subsidence. Therefore, careful selection of the appropriate stem size and precise imaging techniques are crucial for minimizing the risk of experiencing subsidence for patients with obesity.

## Figures and Tables

**Table 1:** The evaluation of demographic data revealed equally distributed patient cohorts.

Variable	Overall	BMI < 30	BMI ≥ 30	*p* Value
No.	163	77	86	
Age (SD)	66.0 (11.1)	67.2 (10.3)	65.0 (11.7)	0.288 *
Sex (%)				0.467 **
Female	84 (51.5)	42 (54.5)	42 (48.8)	
Male	79 (48.5)	35 (45.5)	44 (51.2)	

* Mann–Whitney U test; ** Chi-square test. The data are presented as the mean (SD) for Age and absolute numbers (%) for Sex. SD = standard deviation.

**Table 2:** Statistical analysis of the two groups revealed significantly greater subsidence in the obesity group.

Variable	Overall	BMI < 30	BMI ≥ 30	*p* Value
No.	163	77	86	
Weight [SD]	86.5 (15.8)	75.2 (12.5)	96.5 (11.0)	<0.001 *
BMI [SD]	30.0 (4.4)	26.4 (2.8)	33.3 (2.7)	<0.001 *
Subsidence [SD]	2.4 (2.0)	2.2 (2.1)	2.6 (1.8)	0.015
Harris Hip Score [SD]				
Preoperative	50.7 (18.4)	56.5 (19.2)	45.5 (16.0)	<0.001 *
Postoperative	89.7 (14.9)	94.1 (10.4)	85.6 (17.1)	<0.001 *
Surgery time [SD]	82.1 (27.9)	83.5 (27.2)	80.9 (28.6)	0.452 *
Length of stay [SD]	10.5 (29.2)	12.2 (41.7)	9.1 (8.2)	0.633 *
Blood loss [SD]	840.5 (493.5)	777.5 (433.8)	907.5 (531.9)	0.246 *
CCD [SD]	130.7 (7.0)	131.1 (6.4)	130.3 (7.6)	0.454 *

* Mann–Whitney U test. The data are presented as the mean (SD). SD = standard deviation

**Table 3:** Results of the regression analysis.

Predictors	Estimates	std. Beta	CI	Standardized CI	*p*
(Intercept)	−0.61	0	−3.13–1.91	−0.15–0.15	0.632
Age	0.01	0.06	−0.02–0.04	−0.09–0.22	0.44
BMI	0.03	0.08	−0.03–0.08	−0.09–0.24	0.35
Shaft size	0.19	0.23	0.06–0.32	0.07–0.38	0.005
Deviation	0.42	0.24	0.13–0.70	0.07–0.40	0.005
Observations: 147	R^2^/R^2^ adjusted: 0.131/0.107				

**Table 4:** The planning of the stem seems to be more accurate in the nonobesity group.

		Deviation			Sum
		0	1	2	
BMI < 30	*n*	58	10	9	77
	%	75.32	12.99	11.69	100.00%
BMI ≥ 30	*n*	41	10	35	86
	%	47.67	11.63	40.70	100.00%
Sum	*n*	99	20	44	164
	%	60.74	12.27	26.99	100.00%

0 means that the actual size matches the planned stem size; 1 means a smaller stem than planned was used; 2 means a bigger stem than planned was used.

**Table 5:** Clavien–Dindo classification of postoperative complications [23].

	BMI < 30 (*n* = 77)	BMI ≥ 30 (*n* = 86)
No complication	54 (70.1%)	56 (65.1%)
grade 1	12 (15.6)	11 (12.8)
grade 2	5 (6.5)	5 (5.8)
grade 3	6 (7.8)	9 (10.5)
grade 4	0 (0)	5 (5.8)
grade 5	0 (0)	0 (0)

The data are presented as absolute numbers (%).

## Data Availability

The data presented in this study are available on request from the corresponding author.

## References

[1] Patel I., Nham F., Zalikha A.K., El-Othmani M.M. (2023). Epidemiology of Total Hip Arthroplasty: Demographics, Comorbidities and Outcomes. Arthroplasty.

[2] Singh J.A., Yu S., Chen L., Cleveland J.D. (2019). Rates of Total Joint Replacement in the United States: Future Projections to 2020-2040 Using the National Inpatient Sample. J. Rheumatol..

[3] Singh J.A. (2011). Epidemiology of Knee and Hip Arthroplasty: A Systematic Review. Open Orthop. J..

[4] Leiss F., Götz J.S., Meyer M., Maderbacher G., Reinhard J., Parik L., Grifka J., Greimel F. (2022). Differences in Femoral Component Subsidence Rate after THA Using an Uncemented Collarless Femoral Stem: Full Weight-Bearing with an Enhanced Recovery Rehabilitation versus Partial Weight-Bearing. Arch. Orthop. Trauma Surg..

[5] Jerosch J. (2017). Kurzschaft Ist Nicht Gleich Kurzschaft. Orthopädie Rheuma.

[6] Zimmerer A., Slouka S., Kinkel S., Fritz T., Weiss S., Sobau C., Miehlke W. (2020). Comparison of Short-Stem with Conventional-Stem Prostheses in Total Hip Arthroplasty: An 8-Year Follow-up Study. Arch. Orthop. Trauma Surg..

[7] Haynes J., Nam D., Barrack R.L. (2017). Obesity in Total Hip Arthroplasty. Bone Jt. J..

[8] Hales C.M., Carroll M.D., Fryar C.D., Ogden C.L. (2017). Prevalence of Obesity Among Adults and Youth: United States, 2015–2016.

[9] Obesity and Overweight. https://www.who.int/news-room/fact-sheets/detail/obesity-and-overweight.

[10] Streit M.R., Haeussler D., Bruckner T., Proctor T., Innmann M.M., Merle C., Gotterbarm T., Weiss S. (2016). Early Migration Predicts Aseptic Loosening of Cementless Femoral Stems: A Long-Term Study. Clin. Orthop. Relat. Res..

[11] Schaer M.O., Finsterwald M., Holweg I., Dimitriou D., Antoniadis A., Helmy N. (2019). Migration Analysis of a Metaphyseal-Anchored Short Femoral Stem in Cementless THA and Factors Affecting the Stem Subsidence. BMC Musculoskelet. Disord..

[12] Gabrion M., Rattier S., Blondin E., Michaud A., Mertl P., Gabrion A. (2023). Survival and Radioclinical Evaluation of the Optimys^TM^ Short Stem at More than 6 Years’ Mean Follow-up: A Retrospective Study of 108 Cases. Orthop. Traumatol. Surg. Res..

[13] Kutzner K.P., Kovacevic M.P., Freitag T., Fuchs A., Reichel H., Bieger R. (2016). Influence of Patient-Related Characteristics on Early Migration in Calcar-Guided Short-Stem Total Hip Arthroplasty: A 2-Year Migration Analysis Using EBRA-FCA. J. Orthop. Surg. Res..

[14] Kutzner K.P., Ried E., Donner S., Bieger R., Pfeil J., Freitag T. (2020). Mid-Term Migration Pattern of a Calcar-Guided Short Stem: A Five-Year EBRA-FCA-Study. J. Orthop. Sci..

[15] Kutzner K.P., Donner S., Loweg L., Rehbein P., Dargel J., Drees P., Pfeil J. (2019). Mid-Term Results of a New-Generation Calcar-Guided Short Stem in THA: Clinical and Radiological 5-Year Follow-up of 216 Cases. J. Orthop. Traumatol..

[16] Freitag T., Kappe T., Fuchs M., Jung S., Reichel H., Bieger R. (2014). Migration Pattern of a Femoral Short-Stem Prosthesis: A 2-Year EBRA-FCA-Study. Arch. Orthop. Trauma Surg..

[17] Anderl C., Johl C., Krüger T., Hubel W., Weigert U., Mittelstaedt H., Ortmaier R. (2024). Subsidence after Calcar-Guided Short Stem Total Hip Arthroplasty: Five-Year Results of a Prospective Multicentre Study. Int. Orthop..

[18] Reinbacher P., Smolle M.A., Friesenbichler J., Draschl A., Leithner A., Maurer-Ertl W. (2022). Three-Year Migration Analysis of a New Metaphyseal Anchoring Short Femoral Stem in THA Using EBRA-FCA. Sci. Rep..

[19] Biedermann R., Krismer M., Stöckl B., Mayrhofer P., Ornstein E., Franzén H. (1999). Accuracy of EBRA-FCA in the Measurement of Migration of Femoral Components of Total Hip Replacement. J. Bone Jt. Surg..

[20] Biedermann R., Stöckl B., Krismer M., Mayrhofer P., Ornstein E., Franzén H. (2001). Evaluation of Accuracy and Precision of Bone Markers for the Measurement of Migration of Hip Prostheses. J. Bone Jt. Surg..

[21] Bourke D.L., Smith T.C. (1974). Estimating Allowable Hemodilution. Anesthesiology.

[22] Gross J.B. (1983). Estimating Allowable Blood Loss: Corrected for Dilution. Anesthesiology.

[23] Sink E.L., Leunig M., Zaltz I., Gilbert J.C., Clohisy J. (2012). Reliability of a Complication Classification System for Orthopaedic Surgery. Clin. Orthop. Relat. Res..

[24] Migliorini F., Maffulli N., Pilone M., Velaj E., Hofmann U.K., Bell A. (2023). Demographic Characteristics Influencing the Stem Subsidence in Total Hip Arthroplasty: An Imaging Study. Arch. Orthop. Trauma Surg..

[25] Dammerer D., Blum P., Putzer D., Krappinger D., Liebensteiner M.C., Nogler M., Thaler M. (2022). Subsidence of a Metaphyseal-Anchored Press-Fit Stem after 4-Year Follow-up: An EBRA-FCA Analysis. Arch. Orthop. Trauma Surg..

[26] Saracco M., Fidanza A., Necozione S., Maccauro G., Logroscino G. (2022). Could Short Stems THA Be a Good Bone-Saving Option Even in Obese Patients?. J. Clin. Med..

[27] Mittelstaedt H., Anderl C., Ortmaier R., Johl C., Krüger T., Wallroth K., Weigert U., Schagemann J.C. (2023). Subsidence Analysis of a Cementless Short Stem THA Using EBRA-FCA—A Seven-Year Prospective Multicentre Study. J. Orthop..

[28] Kamiński P., Szmyd J., Ambroży J., Jurek W. (2015). Postoperative Migration of Short Stem Prosthesis of the Hip Joint. Ortop. Traumatol. Rehabil..

[29] Hungerford M.W., Schuh R., O’Reilly M.P., Jones L.C. (2014). Outcome of Minimally Invasive Hip Replacement in Obese, Overweight, and Nonobese Patients. J. Surg. Orthop. Adv..

[30] Afghanyar Y., Danckwardt C., Schwieger M., Felmeden U., Drees P., Dargel J., Rehbein P., Kutzner K.P. (2020). Primary Stability of Calcar-Guided Short-Stem Total Hip Arthroplasty in the Treatment of Osteonecrosis of the Femoral Head: Migration Analysis Using EBRA-FCA. Arch. Orthop. Trauma Surg..

[31] Holzer L.A., Scholler G., Wagner S., Friesenbichler J., Maurer-Ertl W., Leithner A. (2019). The Accuracy of Digital Templating in Uncemented Total Hip Arthroplasty. Arch. Orthop. Trauma Surg..

[32] Hinz N., Marsoni G., Mittelstädt H., Sonnabend F., Wallroth K., Johl C., Weigert U., Anderl C., Ortmaier R., Zeleny N. (2024). Short Stem Hip Arthroplasty with the Optimys Prosthesis Is a Safe and Effective Option for Obese Patients: A Mid-Term Follow-up Multicenter Study. Arch. Orthop. Trauma Surg..

[33] Barrett M., Prasad A., Boyce L., Dawson-Bowling S., Achan P., Millington S., Hanna S.A. (2018). Total Hip Arthroplasty Outcomes in Morbidly Obese Patients: A Systematic Review. EFORT Open Rev..

